# Big Health Data and Cardiovascular Diseases: A Challenge for Research, an Opportunity for Clinical Care

**DOI:** 10.3389/fmed.2019.00036

**Published:** 2019-02-25

**Authors:** Angelo Silverio, Pierpaolo Cavallo, Roberta De Rosa, Gennaro Galasso

**Affiliations:** ^1^Cardiology Unit, Cardiovascular and Thoracic Department, University Hospital “San Giovanni di Dio e Ruggi d'Aragona”, Salerno, Italy; ^2^Department of Physics “E.R. Caianiello”, University of Salerno, Salerno, Italy

**Keywords:** electronic health records, big data, cardiovascular disease, heart failure, acute coronary syndromes, coronary artery disease

## Abstract

Cardiovascular disease (CVD) accounts for the majority of death and hospitalization, health care expenditures and loss of productivity in developed country. CVD research, thus, plays a key role for improving patients' outcomes as well as for the sustainability of health systems. The increasing costs and complexity of modern medicine along with the fragmentation in healthcare organizations interfere with improving quality care and represent a missed opportunity for research. The advancement in diagnosis, therapy and prognostic evaluation of patients with CVD, indeed, is frustrated by limited data access to selected small patient populations, not standardized nor computable definition of disease and lack of approved relevant patient-centered outcomes. These critical issues results in a deep mismatch between randomized controlled trials and real-world setting, heterogeneity in treatment response and wide inter-individual variation in prognosis. Big data approach combines millions of people's electronic health records (EHR) from different resources and provides a new methodology expanding data collection in three direction: high volume, wide variety and extreme acquisition speed. Large population studies based on EHR holds much promise due to low costs, diminished study participant burden, and reduced selection bias, thus offering an alternative to traditional ascertainment through biomedical screening and tracing processes. By merging and harmonizing large data sets, the researchers aspire to build algorithms that allow targeted and personalized CVD treatments. In current paper, we provide a critical review of big health data for cardiovascular research, focusing on the opportunities of this largely free data analytics and the challenges in its realization.

## Introduction

Owing to the aging population, growing urbanization and globalization, cardiovascular disease (CVD) have overtaken communicable diseases as the world's major disease burden (1–3). In spite of the efforts in improving prevention, diagnosis and treatment, CVD represents the leading cause of disability and premature death throughout the world. About 17 millions of death per year are attributed to cardiovascular causes (two times as many deaths as was caused by cancer) and the number is expected to grow to 23.3 million by 2030 (4, 5). The annual estimated cost of CVD in the United States for 2012–2013 was $316.1 billions: $189.7 billions was the direct cost including the expenses of physicians and other professionals, prescribed medication, hospital services, and home health care; $126.5 billions is the indirect cost, comprehensive of lost future productivity attributed to premature CVD mortality or disability (6). In Europe, CVD was estimated to cost €210 billions in 2015: 53% (€111 billions) is due to health care costs, 26% (€54 billions) to lost productivity and 21% (€45 billions) to the informal care of people with CVD (7). The costs to healthcare service vary among countries. In a previous analysis on the economic burden of acute coronary syndromes (ACS) in five European countries, the total cost was €1.9 billions in the United Kingdom (UK), €1.3 billions in France, €3.3 billions in Germany, €3.1 billions in Italy, and €1.0 billions in Spain. Despite differences in the incidence of ACS, these costs reflect variations in the expenditure per ACS patients and were associated to a trend toward lower mortality: €7,009 in the UK, €8,447 in France, €8,280 in Germany, €12,086 in Italy and €9,717 in Spain (8). The costs for CVD are higher than any other group of disease, and their increase in the next years may be offset by an advisable reduction of their incidence due to primary prevention and risk factor control measures.

The CVD global burden counteracts the limited resources for investments in sustainable health and research policies. The deep mismatch between the need for data and the actual resources allocated highlights the importance to develop new forms of cardiovascular research capable of analyzing large amounts of information cost-effectively. Many data are often readily available but, since they were collected for other purposes, are not processed by conventional research practice. Other times, data are part of small or large datasets not linked to each other. The optimization of resources goes through the removal of barriers between data sources and the construction of platforms able to handle such huge amount of informations.

## Big Health Data: What are They?

In the last decade, “big data” has been used to define a research approach involving the use of large-scale, complex datasets (9, 10). Although difficult, it may be defined as a “cultural, technological, and scholarly phenomenon” based on the application of machine learning algorithms to data process and analysis (11, 12). In biomedical research, big data is used for electronic health record (EHR) considered “relevant” to the understanding of health and disease, including clinical, imaging, omic, data from internet use and wearable devices, and others (13). The basic principle is to make the whole biomedical practice “evidence generating” without the need to design and conduct *ad hoc* studies (13, 14). The most popular description for big data was proposed by Doug Laney in 2001 and is known in the academic world as the “3Vs”: volume, variety, and velocity (15).

### Volume

An unimaginable amount of data is created every year and only a very small part is used for research. Since the total amount of data is projected to double every 2 years, in 2020 we are having 50 times more data (44 zettabytes, or 44 trillion gigabytes) than in 2011. To give an idea, 1 kilobyte is the size of a page of text, 1 gigabyte correspond to about 6 millions of books, and a typical large tertiary care hospital generates about 100 terabytes of data per year. This explosion lies in the possibility to store huge quantity of data, as the average price of gigabyte of storage fell in the last 30 years, and easy access to them (16).

High sample size is required to investigate both rare and common CVD (particularly if the endpoint is infrequent). From the end of the Second World War to today, the volume of logs (first in the United States, then in Europe) has been gradually increasing. The Framingham study started in 1948 and enrolled 5,209 patients in its original cohort. Subsequently, the population increased with additional cohorts (off spring, third generation, new off spring spouse, Omni 1 and Omni 2) and to date counts more than 15 thousands subjects (17). In 2014, through a nation-wide enrollment of patients with ischemic heart disease, the SWEDEHEART registry included 105,674 patients with ST-elevation myocardial infarction (STEMI) and 205,693 with non-ST-elevation myocardial infarction (NSTEMI) (18, 19). Other research projects performed a cross-national collection of data such as the PURE study, which included 140 thousands subjects from 21 countries in five continents between 2003 and 2018 (20). Eventually, the CALIBER program for CVD research, by accessing to longitudinal data of linked EHR in UK, assembled a huge population of 1.25 million patients (21).

As the mountains of accumulated cardiovascular data grow, the desire to analyze and convert them into results also grows.

### Variety

Historically, the majority of EHR were structured in spreadsheet or databases. However, the variety of data has become much less congruent and stored in countless forms with a growing trend toward “unstructured” format. Structured data are highly-organized informations easy to process and analyzed (22). Examples of structured CVD data are: age, drug doses, lab values, electrocardiogram (ECG), echocardiographic values, genetic data (e.g., single nucleotide polymorphisms, copy number variations and rare mutations), tools for genetic analysis (e.g., arrays and next generation sequencing), etc. Unstructured data do not have a pre-defined data model or schema, and may be textual or non-textual, human- or machine-generated (22). However, many unstructured informations may be helpful to assemble a holistic view of a patient, including social and environmental factors potentially influencing health. Among them we can find: medical instructions, differential diagnosis, reports, digital clinical notes, physical examination, but also blogs, tweets, and Facebook posting. Most of the Database Management Systems through interchange and translation mechanism allow to overcome the barriers of the past related to the variety of incompatible data formats, non-aligned data, and inconsistent data semantics (15). The technical obstacles in linking such variety of informations is one of the main challenge of big data analytics (22, 23). For example, the absence of a unique patient identifier in the United States has limited the linkage of data for research purpose. However, the development of increasingly sophisticated probabilistic algorithms based on the available demographics data (e.g., name, age, zip code, etc.) allows linking informations with an acceptable risk of error (22).

### Velocity

Big data must provide solutions that reduce the time for storing and retrieving of data packets (so called “data latency”). Velocity, in fact, requires architectures which do not assume that the informations must be near real time. The enterprises have developed solution such as operational data stores which periodically extract and reorganize data for operational inquiry, caches providing instant access to the informations, and point-to-point data routing between apps and databases. Another possible future direction to boost velocity is the application of “anytime algorithms” that can learn from streaming data and that return a valuable result if their execution is stopped at any time (15, 24).

The velocity for creation, storing, and analyzing data is an index of performance of EHR analytics and is essential for its real-world application in cardiovascular research. On the other side, the high speed at which data are generated, increased the gap between the volume of informations available, and our ability to analyze and interpret them.

## Electronic Health Records Sources

The first problem in using big health data is to identify the potential sources of information and to determine the value of linking them together. The common sources for data analysis in cardiology includes the use of conventional datasets such as administrative databases, clinical and population-based registries, and longitudinal cohort datasets. However, the amount of sources available today is truly unimaginable and big data platforms must be ready to welcome new ones as they are rapidly evolving. Additional informations can be received directly from the patients (e.g., health and biometric data from wearable technologies such as heart rate, blood pressure, calories burned, steps walked and time spent exercising) (25), from reports (e.g., health surveys on lifestyle and drug compliance to long-term anti-hypertensive or anti-lipidemic therapy), social media (blogs, tweets, Facebook posting), medical imaging data (echocardiography, cardiac magnetic resonance, cardiac computed tomography, etc.), biomarker data (troponin, brain natriuretic peptide), omics data (including genomic, proteomic, and metabolomic), and others.

Conventional EHR include many types of data (comorbidities, ECG, echocardiography, laboratory values, etc.) providing depth on an individual outpatient visit, hospitalization, etc. Other sources such as claims or insurance data depicts longitudinally the patient's medical history over a definite period, but are limited to few categories (22). Hemingway et al. depicted the “tradeoffs” between depth of informations (registries, EHR, imaging, genomics and multi-omics) and scale (numbers of subjects included), emphasizing such discrepancies among the majority of current data sources relevant for cardiovascular research (13). Despite the challenges in accessing to scale, EHR resources provide a number of phenotypic informations definitely higher than any single registry (26). EHR are myriad by definition and potentially provide detailed informations on demographic and clinical features, instrumental data, etc. Despite many of them were designed to include genomic data, to date the bigger ones (CALIBER, Mondriaan and ABUCASIS) are population-based and do not provide genomic informations. Table 1 reports some well-known EHR resources for CVD research distinguished according to the designs (population-, disease- or hospital-based) and the inclusion or not of genomic data.

**Table 1:** EHR resources relevant for cardiovascular research.

**Name**	**Country**	**Size**	**Website**
**EHR RESOURCES NOT INCLUDING GENOMIC DATA**			
**Population-based**			
CArdiovascular research using LInked Bespoke studies and Electronic health Records (CALIBER)	UK	10,000,000 subjects	https://www.caliberresearch.org
Mondriaan	NE	15,000,000 subjects	https://www.rug.nl/research/portal/publications/the-mondriaan-project-the-dutch-healthcare-landscape-as-a-population-laboratory(5e97d078-e6cc-40a3-9637-dbdd181b14fc).html
ABUCASIS	ES	5,000,000 subjects	http://publicaciones.san.gva.es/prof/calidadyacred/sistemasdeinfo.html
**Hospital-based**			
National Institute for Health Research Health Informatics Collaborative (HIC)	UK		http://www.hic.nihr.ac.uk
**Disease-based**			
SWEDEHEART	SE	2,000,000 subjects	http://www.ucr.uu.se/swedeheart/
European Society of Cardiology European Research Programme (EORP)	EU	2,200 centers 100,000 subjects	https://www.escardio.org/Research/Registries-&-surveys/Observational-research-programme
National Institute for Cardiovascular Outcomes Research (NICOR)	UK	200,000 records on HF 1,000,000 records on CRD 450,000 records on PCI	https://www.ucl.ac.uk/nicor
**EHR RESOURCES INCLUDING GENOMIC DATA**			
**Population-based**			
UK-Biobank	UK	500,000 subjects	http://www.ukbiobank.ac.uk/
European Prospective Investigation into Cancer and Nutrition (EPIC) -CVD	EU	10 countries 500,000 subjects	http://www.epiccvd.eu/
China Kadoorie Biobank	China	500,000 subjects	http://www.ckbiobank.org/site/
UCL-LSHTM-Edinburgh-Bristol (UCLEB) Consortium	UK	30,000 subjects	http://datacompass.lshtm.ac.uk/40/
**Hospital-based**			
US Department of Veteran Affairs-Million Veteran Program	US	500,000 subjects	https://www.research.va.gov/mvp/
Kaiser Permanente-Research Program on Genes, Environment and Health	US	500,000 subjects	http://www.rpgeh.kaiser.org/
eMERGE	US	105,000 subjects	https://emerge.mc.vanderbilt.edu/
DiscovEHR project of the Regeneron Genetics Center and the Geisinger Health System	US	42,000 subjects	http://www.discovehrshare.com
Precision Medicine Initiative Cohort Program	US		https://allofus.nih.gov/
Vanderbilt BioVU	US		https://victr.vanderbilt.edu/pub/biovu/
**Disease-based**			
GENIUS-CHD	Global	250,000 subjects	http://www.genius-chd.com/
HERMES Consortium	Global	30,000 subjects	http://www.hermesconsortium.org/
AFGen Consortium	EU-US		https://www.afgen.org/

*CRD, cardiac rhythm diseases; ES, Spain; EU, Europe; HF, heart failure; NE, Netherlands; PCI, percutaneous coronary intervention; SE, Sweden; UK, United Kingdom; US, United States*.

## Big Health Data Opportunities

Big health data can improve the quality of cardiovascular research in many ways and probably its potential is largely underestimated. Definitely, EHR insights may change patients' care and make more efficient the Health systems. Table 2 reports a list of the main opportunities provided by big data analytics. Here are some of them supported by examples from CVD literature.

**Table 2:** Main opportunities and challenges of EHR for cardiovascular research.

**Opportunities**	
•High-resolution large-scale studies	Large cohorts allow to study infrequent events
•Public health improvement	EHR improve quality of healthcare and spending control
•Development of predictive models	Machine learning predictive models do not require statistical assumption and use complex algorithm for the analysis of large and heterogeneous dataset
•Timely answers to cardiology controversy	Big data provide real-time response to the problems of daily clinical practice
•Drug surveillance	EHR-based post-marketing surveillance of adverse drug events
•International comparison	Assessment of performance of healthcare system of different countries in term of patients' outcome and costs
•Integrating pharmacogenomics	Informatics models by disseminating patient information at the point of care may facilitate the development of pharmacogenomic clinical decision support in daily practice
•Personalized medicine by estimates of benefits and harms of treatments	
•Quality of care and performance measures	Monitoring quality of treatments and support continuous improvement in the participating centers
•Drug repurposing	Data linkage in big dataset may help to identify new uses for approved or investigational drugs that are outside the scope of the original medical indication
•Genetic insights
**Challenges**	
•Disease definition	Heterogeneous and not standardized disease definitions are a challenge for computation
•Source availability	
•Data sharing	The practice of making data used for research available to other investigators.
•Data quality and missing data	
•Translational applicability of results	Apply findings from big data analytics to enhance diagnosis, treatment and prognostic stratification of diseases
•Dependence problem	Situation in which a program instruction is dependent on a result of a sequentially previous instruction before it can complete its execution
•Data linkage	Method of bringing together informations from different sources about the same person or clinical entity
•Data inconsistency	If the same data is stored in different formats in two files and matching of data must be done between files. Moreover, these files duplicate some of the data
•Interpretation of results	
•Unstructured data	Processing data not having a pre-defined model or not organized in a pre-defined manner
•Data integrity	Data integrity is the maintenance of the accuracy and consistency of data over its entire life-cycle
•Training	
•Legal and ethical issues	
•Data security	

*EHR, electronic health records*.

### Large-Scale Studies

Large cohorts were traditionally required to study clinically significant events with low prevalence. Infrequent outcomes such as stent thrombosis in patients with coronary artery disease undergoing percutaneous coronary intervention (PCI) have been effectively explored only through high-resolution large-scale studies (27–29). In comparison with other endpoints (e.g., stent restenosis, new revascularization, death, recurrence of myocardial infarction, bleeding, etc.) the occurrence of stent thrombosis is markedly less common and the majority of randomized controlled trials (RCT) are underpowered to detect significant differences for this feared life-threatening event. The Swedish Angiography and Angioplasty Registry (SCAAR) enrolled all coronary interventional procedures performed in Sweden since 2005 with the registration of stent thrombosis in every patients undergoing subsequent coronary angiography for all previously PCI-treated lesions (30). By using SCAAR dataset, researchers were able to investigate the occurrence of stent-thrombosis in specific PCI settings, and provided milestone evidences on drug eluting stent performance in the last decade (31–33).

Other examples of very large datasets used for clinical and research purposes are provided by electrophysiology. Remote monitoring of implantable cardioverter defibrillator is currently recommended, owing to the capability to reduce the rate of inappropriate shocks and to detect with high sensitivity both symptomatic and asymptomatic atrial fibrillation episodes (34–36). Big data on remote monitoring are collected in large-scale studies such as ALTITUDE and MERLIN, which showed a substantial survival benefit in these patients as compared to non-remote monitored ones (37, 38). Data on heart failure patients undergoing remote monitoring after cardiac resynchronization therapy are also collected and may help to modify disease progression and improve survival. Due to the nature of remote monitoring, data on millions of participants worldwide will be available in the next years with the potential to provide high-resolution results.

### Machine Learning Models

Conventional statistical models have many shortcomings, which may affect their application for the analysis of very big and complex dataset. In fact, they are time-consuming, analyze a limited number of variables included in a database, and require assumptions needing confirmation in clinical practice (10, 39, 40). The term machine/deep learning approach indicates a set of algorithms, which allow identifying features from the data and performing prediction (41). This model integrates conventional statistical tools and allows understanding patterns from large and heterogeneous data through the analysis of complex variables. Since it is based on few assumptions, deep learning method provide more reliable and robust predictions.

Machine learning is distinguished in two main types: supervised and unsupervised. In the first case, the dataset include labeled outcomes and is generally used to predict events or to identify variables associated with the outcome (42, 43). In a study on 94 patients, Sengupta et al. developed a supervised learning for the echocardiographic differentiation of constrictive pericarditis and restrictive cardiomyopathy, and demonstrated that this approach might support the images interpretation and standardize the assessment in this clinical setting (44). In a larger multicenter cohort of 25,775 patients, Kwon et al. internally and externally validated an echocardiography-based machine learning model for prediction of in-hospital mortality in CVD patients, which resulted more accurate than other preexisting predictive models (45).

In the unsupervised learning, there are no labels or annotations and the goal is the evaluation of the relationships between variables or, sometimes, the identification of a hidden structure in the dataset. It is based on complex analytical methods such as clustering, information maximizing component analysis, principal component analysis, topological data analysis, self-organizing maps, etc. Among the countless possible applications, unsupervised deep learning model are widely used to implement computerized image analysis in echocardiography or other cardiovascular imaging modalities (e.g., for left ventricular volumes estimation, left ventricular wall segmentation, etc.) (41).

However, the potential of machine learning models to influence clinical decision making also implies the potential for harm, through the dissemination of misinformation (46). The quality of prediction models depends on the quality of the dataset, and issues involving data inaccuracy, missingness, heterogeneous sources, and selective measurement remain substantial concerns when EHR are used to build prognostic models (47, 48). The risk for harm from insufficiently validated models suggests the need for careful surveillance.

### Timely Answers to Cardiology Controversy

Big data aspires to provide real-time solutions to the problems of daily clinical practice and unsolved disputes. A scholastic case was the uncertain association between varenicline, a partial agonist at the α4β2 nicotinic acetylcholine receptor that has proven effective for smoking cessation, and cardiovascular events. In 2010, by evaluating the efficacy and safety of varenicline vs. bupropion in patients with stable CVD, Rigotti et al. showed higher rates of non-fatal MI, need for coronary revascularization, and peripheral vascular disease among patients receiving varenicline (49). Although not statistically significant, these differences prompted the Food and Drug Administration to issue a drug safety communication about a possible increased risk of cardiovascular events with varenicline assumption. A subsequent meta-analysis of 14 RCT found a slight increased risk of adverse cardiovascular events in varenicline compared with placebo (50) while other study-level analysis failed to detect any significant association (51–53). Owing to the inconsistency of data published until then, Svanstrom et al. analyzed EHR data of about 36,000 subjects treated with varenicline or bupropion from the Danish National Prescription Registry (54). The study results, timely published in 2012, showed no increased risk of major cardiovascular events associated with use of varenicline compared with bupropion for smoking cessation. Owing to the low event rate (7 cases per 1,000 person/year), it would not have been feasible to design a study and collect a similar cohort in such a short time frame. This example shows how time is an essential component of the research and big health data are a formidable source of late-breaking science news.

### International Comparison

The international variation in CVD treatment may influence patients' outcome as well as costs, and represents a relevant target of big data analytics. In a study of aggregated data from national and international registries of 12 European countries, Laut et al. showed a substantial difference in the use of primary PCI for patients with STEMI from 2003 to 2008 (55). The authors hypothesized that such variability could have been associated with supply factors, such as numbers of beds and physicians, and to healthcare economic characteristics. However, the absence of a patient-level dataset did not allow to evaluate the influence of patient-related factors (55). The nation-wide collection of data provides the unique opportunity to compare the care system performance of different countries and evaluate the effects of patients' characteristics on outcome. To date, Sweden and UK are the only two countries in the world collecting data on ACS on a national scale. Through the assessment of 119,786 patients in Sweden and 391,077 in the UK hospitalized for acute myocardial infarction, Chung et al. showed a significantly lower mortality at 30-days follow-up after discharge in Sweden as compared to UK (7.6% vs. 10.5%) (56). Such difference emerged as the consequence of an earlier and more extensive use of primary PCI as well as of the greater use of β-blockers at discharge in Sweden. Beyond the relevance of the individual study results, this manuscript demonstrates the importance of international comparison research that may be helpful for the improvement of national health systems and patients' outcome. In absence of national registries in most of the world countries, EHR analytics can fill the gap allowing comparison between them.

### Quality of Care and Performance Measures

For several decades, international societies such as the European Society of Cardiology, the American Heart Association and the American College of Cardiology have been publishing guidelines with recommendation for diagnosis and treatment of CVD. However, there are still variations between different hospitals and regions with regard to the utilization of diagnostic tests, drugs prescription, percutaneous and surgical interventions, which may have consequences for quality of care and health systems costs (57, 58).

Many registry extensively used for research purposes were primarily designed to support the development and uniform use of evidence-based therapies in a region or country. The Swedish cardiovascular National Quality Registries aimed to register changes in the quality and content of patient care over time, to monitor quality of treatments and to support continuous improvement efforts in all participating centers. The individual hospitals as well as health professionals can also compare their results with the average for other centers/operators in the whole of Sweden. Recently, Swedish cardiovascular National Quality Registries were implemented with quality indices which reflect the whole chain of care and raised a great interest from the physicians, decision-makers, the general public, and the media (59).

## Big Health Data Challenges

Despite the huge theoretical potential, the evidences that big data will translate into better patient outcomes are currently very poor. To make order in this arduous scenario, it is necessary to distinguish different types of challenges facing the implementation of big data in cardiovascular care (Table 2). Here are some examples of such complexity.

### Missing Data

The amount of missing data in a database, often not missing at random, may interfere with the analysis or make it invalid. For example, data on serum uric acid are largely missing in the Framingham Heart Study, making analysis difficult and questionable (60). Since informations in EHR are non-systematically collected, the amount of missingness is generally high due to a variety of reasons: some data are omitted by clinician because judged not necessary; refusal by the patient; the subject is unable to attend the data collection. Each statistical method for analyzing missing data has its assumptions and limitations, and sometimes the problem of missing data cannot be solved. By evaluating data for serum cholesterol in 28 cohort studies, Barzi and Woodward reported that missing values could be managed with commonly used statistical methods if fewer than 10%. In studies with 10–60% missing data, substantial differences among methods existed; if missingness were more than 60%, no statistical technique could produce plausible results (61).

Complete-case analysis uses only data from patients with complete records for all visits and ignores patients with any missing data. The available-case analysis, conversely, excludes all missing data and analyzes data as they are. Owing to the nature of EHR, these methods are rarely applicable and alternative analyses for handling the high number of missing values are preferred:

- Imputation techniques (mean imputation, hot-deck imputation, regression imputation, multiple imputation): replace missing data with substituted values;
- Mixed effects regression model: based on likelihood-based method for which the model assumes a specific statistical distribution (such as Normal or Poisson) for the data;
- Generalized estimating equations: similar to mixed effects regression models, assume a model for the mean of the longitudinal measures (without including patient-specific effects) and a correlation matrix for the repeated measures;
- Inference (pattern mixture models and selection models): based on factorization of the joint distribution of measurement and drop-out mechanisms.

Detailed description of statistical techniques for handling missing data are reported elsewhere (62).

### Selection Bias

Big data analyses are basically observational, and thus share some limitations of this kind of studies.

Large-scale EHR allow to overcome the sample size limit observed in many clinical researches, but expose to the risk of systematic error in the treatment effect estimate (63). Subjects included in a dataset may be different for geographic, insurance, medical history profiles, etc. Thus, patients receiving two different treatments may have different distributions of a variable that is associated to an outcome of interest. Big data analytics exploits many statistical techniques to address the problem of confounding such as propensity score analysis, instrumental variable analysis and Mendelian randomization, which is a variant instrumental variable analysis used in genetic studies (64). However, many authors emphasize that results of observational research are primarily hypothesis-generating due to the risk of selection bias, and should not influence clinical practice until their hypotheses are confirmed in adequately powered RCT (64). Tai et al. compared the findings of the Nurses' Health Study (NHS), one of the longest and largest observational study, with the results of RCT reporting the same health outcomes. They found that only few of the associations observed in the NHS have been tested in RCT, and where they have, the agreement between their results was poor (≤ 25%) (65). In fact, a large volume does not necessarily implies a representative sample, which is a requisite for any valid inference, and may generate a number of false positive results.

### Data Analysis and Training

For small datasets, a single test may be powerful enough to reject a null hypothesis (66, 67). However, EHR sources generally include very large dataset with multiplicity of data requiring multiple analyses to establish the significance of a hypothesis and identify correlations (68, 69). Big data analytics very often uses algorithms with multiple testing (logistic regression, latent class analysis, principle component analysis, Bayesian analysis, logarithmic and square-root transformations, classification and regression trees, decision trees, neural networks, etc.) which have been implemented to face such complexity.

Unfortunately, analyses of large datasets are still often suboptimal due to the researcher's lack of knowledge of the available statistical and methodological tools (70). In fact, few clinicians and researchers received a formal training in informatics, coding, data analysis, or other increasingly relevant skills to handle very large databases. On the other hand, algorithms to process and analyze big health data are underdeveloped to some extent and require additional efforts for their implementation (60).

### Interpretation and Translational Applicability of Results

One of the problems of big health data is just the possibility to integrate the output of the analysis in daily cardiology practice. Many translational studies designed to answer questions of interest in healthcare are not self-explanatory due to complexity and inadequate description of the dataset variables and associated metadata (71). For example, the interpretation of analysis output may be biased by subjective assumptions and/or manipulations by analysts, or the quality of data is questionable and does not allow unequivocal conclusions.

### Privacy and Ethical Issue

Privacy is fundamental for individuals and groups to assert and maintain their identity. People preserve their privacy by controlling access to their data. The respect of privacy reflects the respect for people as individuals and access or disclosure of their personal data against their wishes may affect personal well-being and rights.

Data from server can be a target for cybercriminals who can identify or encrypt demographic, social, medical or other individual informations for illegal purposes. Despite the probability that attempts of re-identification succeed are very low (around 0.01%), this risk is not negligible (72).

Any challenge or breach of the normal expectations of privacy, however, should be balanced with the potential benefits of data sharing for the entire community. Use of broad consent models or a “new social contract” for data utilization along with the non-stop search for solutions to implement data security systems, may help to preserve people privacy.

## Conclusions

**Figure 1:**
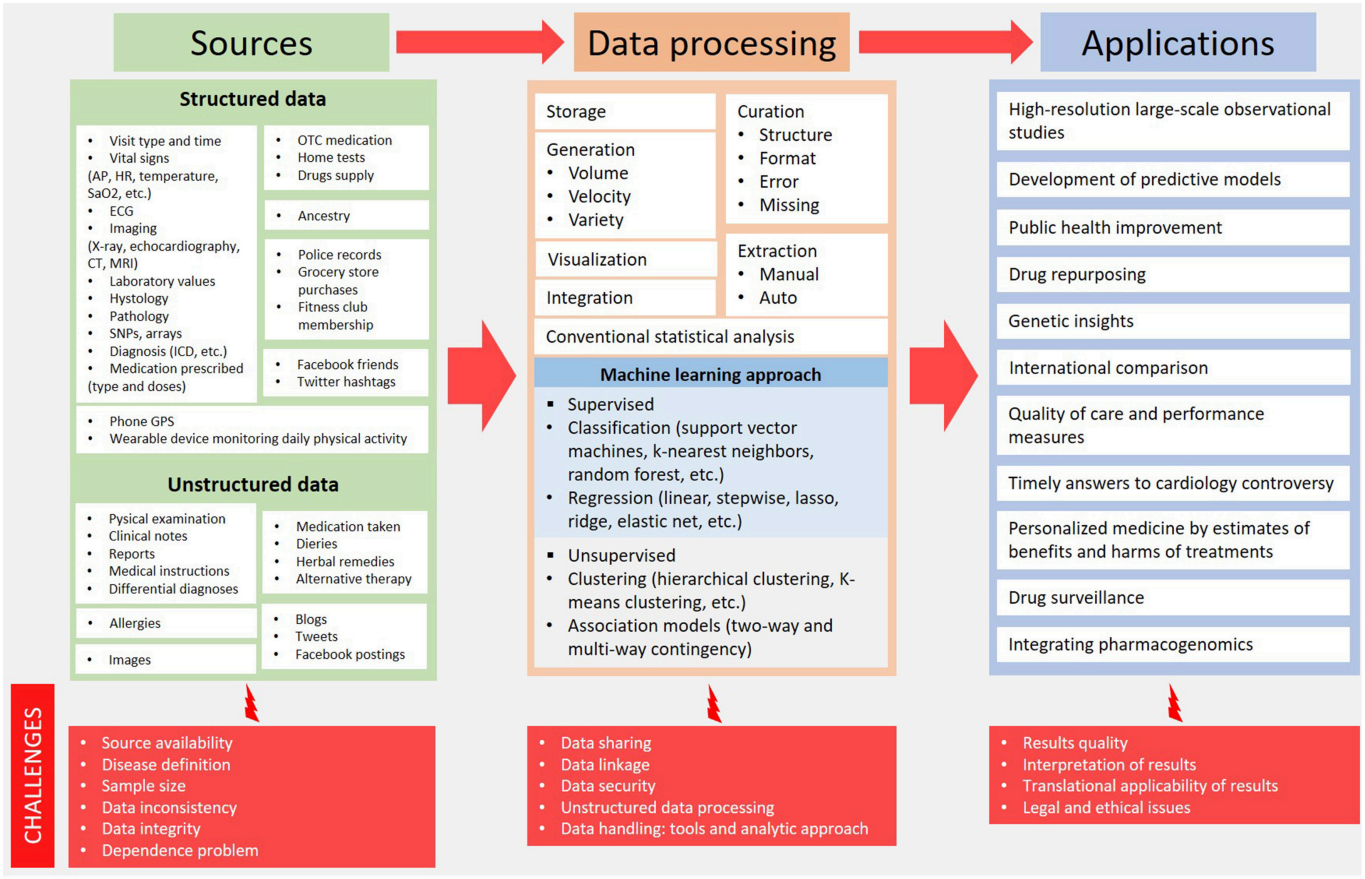
Big health data overview: from sources to potential clinical application. AP, arterial pressure; CT, computed tomography; ECG, electrocardiography; HR, heart rate; ICD, implantable cardioverter defibrillator; MRI, magnetic resonance imaging; OTC, over the counter; SaO_2_, arterial oxygen saturation; SNPs, single nucleotide polymorphisms.

Big health data upsets the traditional research methodologies and provides new ways to get results from a huge amount of sources. Challenges are many and different with each step: source identification, data processing and clinical application of results (Figure 1).

Further efforts are required to move from the mere existence of big data to their widespread use, in order to get a better understanding of CVD. The potential of EHR to guide patient care and improve the efficiency of health systems is unimaginable.

## Author Contributions

All authors listed have made a substantial, direct and intellectual contribution to the work, and approved it for publication.

### Conflict of Interest Statement

The authors declare that the research was conducted in the absence of any commercial or financial relationships that could be construed as a potential conflict of interest.
